# The N-terminal α helix domain of the mitochondrial VDAC protein Por2 is dispensable for promoting the nuclear localization of yeast AMPK

**DOI:** 10.17912/micropub.biology.001040

**Published:** 2026-02-23

**Authors:** Kerry Brown, Hemanth Singuluri, Frank Perkins, Sergei Kuchin

**Affiliations:** 1 Department of Biological Sciences, University of Wisconsin–Milwaukee, Milwaukee, Wisconsin, United States

## Abstract

In yeast, the mitochondrial voltage-dependent anion channel (VDAC) proteins Por1 and Por2 play regulatory roles in the regulation of Snf1, an ortholog of AMP-activated protein kinase (AMPK). An important question is whether Por1 and Por2 serve as Snf1-coupled energy sensors. VDACs are β-barrel proteins, but they have a flexibly-linked N-terminal α helix (NAH) domain, suggesting a possible role in Snf1 signaling. Here, we asked whether the NAH domain of Por2 is required for promoting Snf1 nuclear translocation. In our experimental setup, the Por2 NAH was dispensable. Further experiments are required to fully understand the regulatory roles of the Por1/2 NAH domains.

**Figure 1. Deletion of the Por2 NAH does not affect transcription activation by LexA-Snf1-G53R:**
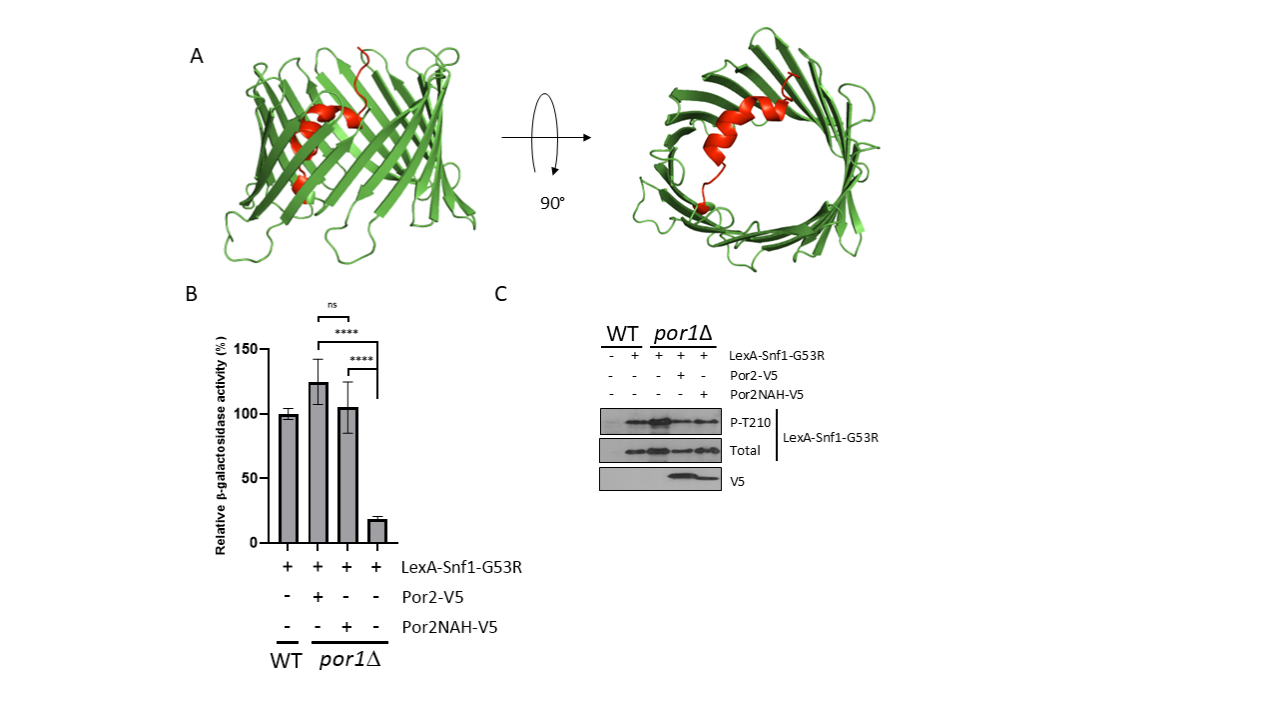
(A) Predicted structure of yeast VDAC Por2 (Uniprot P40478) using AlphaFold. The VDAC core is composed of antiparallel beta strands (green). The flexibly-linked NAH domain is shown in red. (B) WT cells and *por1* ∆ mutant derivative of the reporter strain CTY10-5d expressing LexA-Snf1-G53R and overexpressing either full-size Por2-V5 or Por2NAH-V5, or carrying the corresponding empty vector, were grown in selective SC medium containing high (2%) glucose to mid-log phase and then shifted for 3h to an otherwise identical medium containing low (0.05%) glucose. β-galactosidase activity was assayed in permeabilized cells and measured in Miller units (>5 independent transformants per genotype/plasmid combination). Shown are the values under low-glucose conditions expressed as a percentage of the mean value for wild-type cells carrying the empty vector; in high glucose, all values were less than 0.5% of this reference value. (C) Transformants were shifted to 0.05% glucose as described above and tested for Thr210 phosphorylation of the Snf1-G53R moiety (P-T210), total LexA-Snf1-G53R protein (Total), and Por2-V5 or Por2NAH-V5 (V5) levels by immunoblotting. Error bars indicate standard errors. Statistical analyses were conducted using a two-tailed *t* test with unequal variances. ****, *P* < 0.001; ns, not significant ( *P* > 0.05).

## Description

The AMP-activated protein kinase (AMPK) is conserved in eukaryotes from yeast to humans (Hardie & Carling, 1997). As its name suggests, the key role of AMPK is to respond to reduction in energy levels. When energy levels drop, AMPK is activated to restore the energy balance by inhibiting ATP consumption and promoting ATP generation. AMPK exerts its regulatory effects on various levels, from transcriptional regulation to post-translational effects on protein function (Hardie, 2007).

The AMPK ortholog in budding yeast ( *Saccharomyces cerevisiae* ) is called Snf1 (sucrose-non-fermenting 1) (Hedbacker & Carlson, 2008). Snf1 is required for the utilization of alternative carbon/energy sources when cells experience a shortage of the preferred source – glucose (Hedbacker & Carlson, 2008).

Like mammalian AMPK, the yeast Snf1 kinase complex has a heterotrimeric αβγ structure, where the α subunit is catalytic, and the β and γ subunits are regulatory (Hedbacker & Carlson, 2008). *S. cerevisiae* has a single α subunit (Snf1), a single γ subunit (Snf4), and three alternative β subunits (Sip1, Sip2, Gal83); the three β subunits define three isoforms of the Snf1 complex, which we will refer to as Snf1-Sip1, Snf1-Sip2, and Snf1-Gal83 (Hedbacker et al., 2004). The present work concerns the Snf1-Gal83 isoform.

When yeast cells experience energy stress caused by glucose deprivation, two major events occur. First, the α subunit becomes catalytically activated by phosphorylation of the conserved T-loop threonine residue (Thr210) by upstream kinases (Hong et al., 2003; McCartney & Schmidt, 2001; Sutherland et al., 2003). Second, the Snf1-Gal83 isoform translocates to the nucleus (Vincent et al., 2001). Because Gal83 is normally the most abundant β subunit, Snf1 becomes effectively enriched in the nucleus (Vincent et al., 2001), putting it in a better position to regulate transcription (Abate et al., 2012; Kuchin et al., 2000; Vincent et al., 2001; Young et al., 2003; Young et al., 2012).

The signaling mechanisms behind the Gal83-driven nuclear translocation of the Snf1-Gal83 complex are not completely understood. Previous studies showed that the voltage-dependent anion channel (VDAC) proteins Por1 and Por2 both interact with and contribute to the positive regulation of Snf1 (Strogolova et al., 2012). More recently, we presented evidence that Por1 and Por2 play a positive regulatory role in this process through the Gal83 subunit, further suggesting a role in energy sensing (Shevade et al., 2018).

VDAC proteins, which are highly conserved among eukaryotes, are characterized as having a β barrel structure with a flexible-linked N-terminal α helix (NAH) domain (Blachly-Dyson et al., 1993; Di Rosa et al., 2021; Guardiani et al., 2018). This domain is believed to be able to expose itself to the cytosol, allowing interaction with other proteins or small molecules such as energy-carrying nucleotides, putting VDACs in an advantageous position to serve as energy sensors (Geula et al., 2012; Manzo et al., 2018; Rostovtseva & Colombini, 1997). Here, we have tested for a possible role of the NAH domain of Por2 in promoting Snf1-Gal83 nuclear localization in response to energy stress. The predicted structure of Por2 is shown in Figure 1A .

The *por2* Δ mutation alone does not affect growth on nonfermentable carbon sources and was believed to have no channel activity (Blachly-Dyson et al., 1997). Since its mutation does not hinder respiration, focusing on Por2 allows an opportunity to observe only the regulatory effects of this VDAC. However, there is more recent evidence that Por2 does provide permeability to some ions when incorporated into artificial membranes (Guardiani et al., 2018). While the physiological relevance of these observations remains to be fully investigated, we note that whether or not Por2 is a true channel does not exclude the possibility that it serves as a sensor, by analogy to other transporters/receptors collectively referred to as “transceptors” (Steyfkens et al., 2018). For example, the Mep2 ammonium permease of *S. cerevisiae* also serves as a high-affinity ammonium sensor (Lorenz & Heitman, 1998). While proteomic studies have shown that Por2 protein abundance substantially lower than that of Por1 (Morgenstern et al., 2017), We previously found that cells with a *por1* Δ *por2* Δ double mutation experience a defect in Snf1 threonine-210 phosphorylation (catalytic activation), and this defect was not present in cells with either single mutation alone, indicating that Por1 and Por2 both play redundant roles in Snf1 signaling (Strogolova et al., 2012).

Interestingly, the *por2* ∆ mutation has little, if any, effect on Snf1-Gal83 nuclear localization (Shevade et al., 2018). However, our results showed that overexpression of a C-terminally tagged Por2-V5 protein effectively compensates for the Snf1-Gal83 nuclear enrichment defect caused by the *por1* ∆ mutation (Shevade et al., 2018). While Por2, like other mitochondrial porins, is translated in the cytosol before being translocated to the mitochondria through the TOM complex (Sakaue et al., 2019), it is possible that a fraction of this protein remains cytosolic. We do not know which fraction – mitochondrial or cytosolic – is physiologically relevant for Snf1 nuclear localization, but overexpressed Por2-V5 is clearly functional (Shevade et al., 2018). Therefore, in our experimental setup we compared the ability of full-length Por2 and its N-terminally truncated derivate (Por2∆NAH) lacking amino acids 1-22 to suppress the *por1* ∆ mutation. As done previously, Por2 and Por2∆NAH were expressed with C-terminal V5 tags (Por2-V5 and Por2∆NAH-V5, respectively) from the *ADH1* promoter in multicopy vector pSK71 (Shevade et al., 2018). It is possible that the expression of the native Por2 protein is regulated by physiological conditions, but studying such regulation was not the purpose of this work. Instead, we were interested in the role of the NAH domain. Thus, the use of the strong ADH1 promoter was to ensure the constitutive expression of the Por2-V5 and Por2ΔNAH-V5 proteins.

To determine the effect of the NAH deletion, we used the “shortcut” reporter assay (Kuchin et al., 2000). In this assay, a hyperactive mutant version of Snf1 with a Gly53-to-Arg substitution (Snf1-G53R) is fused to the LexA protein (LexA-Snf1-G53R) and tested for the ability to activate a *lexAop-lacZ* reporter gene containing LexA DNA-binding sites ( *lexAop* ) upstream of *lacZ* (Kuchin et al., 2000). Importantly, the ability of LexA-Snf1-G53R to activate the *lexAop-lacZ* reporter is absolutely dependent on nuclear localization, making it a sensitive quantitative readout of Snf1-Gal83 nuclear translocation in response to carbon/energy stress (Hedbacker et al., 2004; Shevade et al., 2018; Vincent et al., 2001).

Since Por2 overexpression fully compensates for the lack of Por1 (Shevade et al., 2018), we focused on determining whether deletion of the NAH will affect the compensatory ability of Por2 in a *por1* Δ mutant. We overexpressed the Por2-V5 and Por2∆NAH-V5 proteins in the *lexAop-lacZ* reporter strain CTY10-5d (see Materials and Methods)(Shevade et al., 2018) expressing LexA-Snf1-G53R from plasmid pRJ216 (Kuchin et al., 2000). The cells were first grown to mid-log phase with plasmid selection in a medium containing abundant (2%) glucose and then subjected to carbon/energy stress by shifting to an otherwise identical medium containing low (0.05%) glucose for 3h (Kuchin et al., 2000). Assays of β-galactosidase activity in the stressed cells revealed no substantial difference between the ability of overexpressed Por2-V5 and Por2∆NAH-V5 to suppress the *por1* ∆ mutation ( Figure 1B ). Immunoblot analysis indicated that the levels of Por2-V5 and Por2∆NAH-V5 proteins were comparable ( Figure 1C ). The levels of LexA-Snf1-G53R expression and Thr210 phosphorylation (activation) of the Snf1-G53R moiety were comparable as well ( Figure 1C ). Thus, these results provide evidence that the NAH domain of Por2 is dispensable for its ability to promote the nuclear translocation of the Snf1-Gal83 isoform in response to carbon/energy stress.

In conclusion, we note that our results do not automatically mean that the NAH domain of Por2 plays no role at all. For example, its function might be redundant with that of other region(s) of this protein involved in interacting with energy-carrying molecules. In addition, our previous results indicate that besides Snf1-Gal83 nuclear localization, Por2 (together with Por1) plays a distinct role in the catalytic activation of Snf1 (Shevade et al., 2018; Strogolova et al., 2012), and its NAH domain could play a role in that process. Finally, our results with Por2 should not be automatically extrapolated to its paralog Por1 because paralogous proteins often exhibit a degree of functional specialization, implying that their domains might have divergent specialization. Thus, further experiments are required to better understand the signaling roles of eukaryotic VDAC proteins, including the yeast Por1/2 proteins and their NAH domains.

## Methods

Predicted structure of Por2

Amino acid sequence of Por2 was obtained from Uniprot (https://www.uniprot.org/uniprotkb/P04840). The predicted structure was generated using AlphaFold (https://alphafold.ebi.ac.uk/) (Jumper et al., 2021; Varadi et al., 2021).

Yeast strains and growth conditions

The *por1* ∆ mutant strain carrying an integrated *lexAop-lacZ* reporter was described previously (Shevade et al., 2018); it is a derivative of strain CTY10-5d ( *MAT* **a** *gal4 gal80 URA3::lexAop-lacZ his3 leu2 ade2 trp1* ) (R. Sternglanz, SUNY, Stony Brook, NY) (Bartel et al., 1993). Synthetic complete (SC) medium lacking appropriate supplements was used to select for plasmids (Rose MD, 1990). All transformations were performed using standard methods (Rose MD, 1990). Unless indicated otherwise, all yeast media contained 2% glucose, and cells were grown at 30°C.

Plasmids

Plasmid pRJ216 (Kuchin et al., 2000) expresses LexA-Snf1-G53R from the yeast *ADH1* promoter of multicopy vector pEG202 (Golemis et al., 1999). The multicopy vector pSK71 (Shevade et al., 2018) provides expression from the yeast *ADH1* promoter and was constructed by removing the LexA coding sequence from pBTM116 (Fields & Song, 1989). pAMS10 expresses a full-size C-terminal V5 epitope-tagged Por2 from vector pSK71 (Shevade et al., 2018). pFP1 expresses a C-terminal V5 epitope-tagged Por2ΔNAH from vector pSK71 and was constructed as follows. Primers with flanking BamHI sites targeting the *POR2* coding region containing base pairs 69-846 were used to amplify *POR2* Δ *NAH* by PCR from yeast genomic DNA. The resulting PCR fragment was digested with BamHI and inserted into the BamHI site of pSK71.

Assays of *lexAop-lacZ* reporter activation by LexA-Snf1-G53R.

Cells of strains carrying the integrated *lexAop-lacZ* reporter and expressing LexA-Snf1-G53R were grown in appropriate selective SC medium containing high (2%) glucose to mid-log phase and then shifted for 3 h to an otherwise identical medium containing low (0.05%) glucose. Assays of β-galactosidase activity were performed in permeabilized cells and measured in Miller units as described previously (Kuchin et al., 2000).

Immunoblot analysis.

Cells were grown under conditions specified in the text. Protein extracts were prepared by the boiling/alkaline treatment method as described previously (Orlova et al., 2008) and analyzed by immunoblotting. The LexA-Snf1-G53R fusion protein was detected with anti-LexA antibody (MilliporeSigma). The Thr210 phosphorylation state of LexA-Snf1G53R was analyzed using anti-phospho-Thr172-AMPK antibody (Cell Signaling Technology), which strongly recognizes the Thr210-phosphorylated form of yeast Snf1 (Orlova et al., 2008). Signals were detected by enhanced chemiluminescence using the Pierce ECL2 or ECL systems (Thermo Scientific).

## Reagents

Table 1. Strain used in this study.

**Table:** 

Strain	Genotype	Reference
CTY10-5d	*MAT* **a** *gal4 gal80 URA3* :: *lexAop-lacZ his3 leu2 ade2 trp1*	R. Sternglanz, SUNY, Stony Brook, NY
AMS293	CTY10-5d *por1* Δ:: *KanMX6*	Shevade et al., 2018

Table 2. Plasmids used in this study.

**Table:** 

Plasmid	Genotype	Description	Reference
pRJ216	*lexA-SNF1-G53R*	Expresses the LexA-Snf1-G53R fusion protein	Kuchin et al., 2000
pSK71	2µ, *TRP1, ADH1* promoter	Empty vector	Shevade et al., 2018
pAMS10	*POR2-V5*	pSK71-derived expressing the Por2-V5 fusion protein	Shevade et al., 2018
pFP1	*POR2ΔNAH-V5*	pSK71-derived expressing the Por2ΔNAH-V5 fusion protein	This study

Table 3. Primary antibodies used in this study.

**Table:** 

Antibody	Source (catalog number)
Anti-LexA	MilliporeSigma (06-719)
Anti-Phospho-AMPK	Cell Signaling Technology (2325)
Anti-V5	Thermo Scientific (MA1-81617)
